# Self-help support: The Alzheimer’s telephone from the user’s perspective

**DOI:** 10.3205/000331

**Published:** 2024-04-24

**Authors:** Alexander Kurz, Ina Maria Fischer, Vildan Dogan, Carolin Kurz

**Affiliations:** 1 Department of Psychiatry and Psychotherapy, School of Medicine, Technical University of Munich, Germany; 2 Department of Psychiatry and Psychotherapy, LMU University Hospital, LMU Munich, Germany

**Keywords:** dementia care, telephone counseling, German Alzheimer’s Association, quality assessment

## Abstract

**Background:**

Telephone counseling is an important form of support for informal carers of persons with dementia. The quality and benefit of this kind of service have rarely been evaluated in Germany.

**Methods:**

We developed a survey to assess the quality of telephone counseling. We conducted an online survey among 201 users of the telephone hotline “Alzheimer-Telefon” (Alzheimer’s telephone service) provided by the German Alzheimer’s Association after the consultation. The aim of the study was to determine whether this form of telephone support meets certain quality criteria and the callers’ needs.

**Results:**

Of the 201 participants, 80% were female. The mean age of the callers was 51 years. 74% of cases were one-off consultations; 26% of the callers sought advice twice or more often. The most common reasons for calling included behavioral changes (45%) and finding a nursing home (41%). Other family members were significantly (p=0.036) more likely to seek local respite options. Based on the 201 online questionnaires evaluated, most callers were highly satisfied with the counseling services provided by the Alzheimer’s telephone service. Those seeking advice were particularly satisfied with the appreciative and empathetic communication style of the advisors and their professional competence. This also applies to the accessibility of the telephone. More than three quarters were fully satisfied with the information they received. Almost half of the callers were sure that the advice would help to solve their issue. 14% of people seeking advice were uncertain about how to implement the suggested solutions.

A further survey would be worthwhile to determine to what extent the topics of the consultation can be implemented. The feedback from relatives who use the Alzheimer’s telephone repeatedly could be used for this purpose – the repetition rate is currently 25% and the trend is rising. Results could be interesting for successful counseling and for the development of further support services.

**Conclusion:**

The telephone hotline is a useful component of dementia care in Germany and an important contribution to the National Dementia Strategy.

## Zusammenfassung

**Hintergrund:**

Zu den Unterstützungsmöglichkeiten für Angehörige von Menschen mit Demenz zählt die telefonische Beratung. Seit 2002 bietet die Deutsche Alzheimer Gesellschaft Selbsthilfe Demenz e.V. mit ihrem Alzheimer-Telefon telefonische Beratung rund um Demenz kostenlos und bundesweit an. Qualität und Nutzen solcher Dienste wurden hierzulande bisher selten untersucht.

**Methoden:**

Die vorliegende Studie zur kostenlosen Beratung am Alzheimer-Telefon der Deutschen Alzheimer Gesellschaft untersuchte, inwieweit die Beratungsleistung Qualitätskriterien telefonischer Beratungsangebote sowie den Erwartungen und Bedürfnissen der Ratsuchenden entspricht. Hierzu wurde ein Online-Fragenkatalog konzipiert, auf den die Anrufenden im Nachgang an das Beratungsgespräch Zugriff hatten.

**Ergebnisse:**

Von den 201 Teilnehmer:innen waren 80% weiblich; das Durchschnittsalter der Anrufer lag bei 51 Jahren. Bei 74% der Fälle handelte es sich um einmalige Beratungen, 26% der Anrufer suchten zweimal oder öfter Rat. Zu den häufigsten Gründen für den Anruf gehörten Verhaltensänderungen (45%) und die Suche nach einem Pflegeheim (41%). Andere Familienmitglieder waren signifikant (p=0.036) häufiger auf der Suche nach örtlichen Betreuungsmöglichkeiten. Auf Basis der 201 ausgewerteten Online-Fragebögen ergab sich eine hohe allgemeine Zufriedenheit der überwiegenden Mehrheit der Anrufenden mit den Beratungsgesprächen. Insbesondere mit dem wertschätzenden und empathischen Kommunikationsstil der Berater:innen sowie deren fachlicher Kompetenz waren die Ratsuchenden besonders zufrieden. Dies galt ebenso für die Erreichbarkeit des Telefons. Die Erwartungen der Anrufer:innen an den Anruf waren breit gestreut und reflektierten die vielfältigen Lebensbereiche, die im Rahmen einer Demenz betroffen sein können. Mehr als 75% der Anrufenden waren mit der Qualität der erhaltenen Informationen voll zufrieden. Knapp die Hälfte der Anrufenden waren sich sicher, dass die Beratung zur Lösung ihres Anliegens beitragen würde. 14% der Ratsuchenden war sich jedoch unsicher, inwieweit es ihnen gelingen würde, die besprochenen Lösungswege umzusetzen. Was es zusätzlich braucht, um die in der Beratung besprochenen Entlastungs- und Lösungsvorschläge umzusetzen, wäre eine weitere Erhebung wert. Untersucht werden könnten dafür die Rückmeldungen von Angehörigen, die das Alzheimer-Telefon wiederholt nutzen – die Wiederholungsrate liegt derzeit bei 25%, Tendenz steigend. Dies könnte interessant sein sowohl für die Beratung als auch für die Weiterentwicklung entsprechender Unterstützungsangebote. Verbesserungsmöglichkeiten des Alzheimer-Telefons könnten in dem verstärkten Angebot von Folgegesprächen, einem Fokus auf Angehörige mit besonderem Unterstützungsbedarf (bspw. frühe Diagnose oder nicht verwandte Personen, Personen mit Migrationshintergrund) und der Intensivierung der Zusammenarbeit mit nachgeschalteten Beratungsformen am Wohnort der Anrufenden bestehen.

**Schlussfolgerung:**

Die telefonische Beratung ist ein nützlicher Baustein der Demenzversorgung in Deutschland und ein wichtiger Beitrag im Rahmen der Nationalen Demenzstrategie – insbesondere im Hinblick auf den besonderen Bedarf an Information und Beratung von Angehörigen mit einer Demenzerkrankung.

## 1 Introduction

The prevalence of people with dementia in Germany is currently estimated at around 1.6 million, with an incidence of 300,000 new cases per year [1]. About three quarters of people with dementia in Europe are cared for by family members or other close relatives [2]. Family carers are mostly partners (46%) or children (45%), the majority are women (80%), the average age is 64 years, and one third of family carers are employed [3], [4]. The proportion of single people – especially single women – with dementia can be expected to increase in the coming years due to divorces, emigration and low birth rates. This means that an increasing number of unrelated or distant relatives will be caring for a person with dementia [2]. The average amount of time spent caring is 7.5 hours per day; 66% of relatives care for their relative for one year, 33% for more than five years [5], [6]. Caregivers take on significant psychological, social, organizational and financial burdens. Often, these burdens have a negative impact on their own physical and mental health [7], [8]. Primary burden factors include the duration and time required to care for relatives, as well as frequent neuropsychiatric symptoms of dementia such as apathy, aggression, agitation, disinhibition and psychotic experiences [5]. Carers of people with dementia are at a significantly greater risk of developing depression, anxiety, dependence or pain than the general population, and are also have a significantly greater risk of cardiovascular disease [9]. Family members’ mental and physical health directly affects people with dementia [9]. However, not all family caregivers become ill themselves; coping with family caregiving must be understood as a complex adaptation process. In this process, knowledge about the disease and existing support, the use of problem-solving strategies, and a perception of self-efficacy are important factors in coping positively with the caregiving responsibilities of a person with dementia [9]. Family caregivers need to be informed, counseled and supported [10]. The family doctor’s office is usually the first point of contact with the health and social care system, so doctors need to be well informed about the care options available [11]. Telephone helplines provide an important source of support for family caregivers [12]. They are free of charge, easy to access, and flexible in terms of time, and could be used during the Covid-19 pandemic as well [13], [14]. They meet important information needs, reduce depressive symptoms, inform and motivate people to make use of face-to-face support and counseling services and facilitate the search for appropriate resources [15], [16]. The quality and benefits of telephone-based dementia-related counseling have rarely been investigated in Germany [17].

The nationwide “Alzheimer-Telefon” (Alzheimer’s telephone service – ATS) of the German Alzheimer’s Society receives around 5,500–6,000 calls and e-mail inquiries every year and has been funded by the Federal Ministry for Family Affairs, Senior Citizens, Women and Youth since 2002 [18]. The range of advice is broad and includes information on the causes, forms, diagnosis and treatment options for dementia. Relatives also receive basic knowledge on communication and how to deal with challenging behavior. The advice also covers legal and financial issues as well as support options at home. In 90% of cases, these are one-off consultations; around 10% of those seeking advice call twice or more often. In addition to their professional qualifications (social work, social science, psychology or mental health), some members of the counseling team also have experience as family caregivers. The quality of advice is ensured through regular team meetings, continuous supervision and external training. The National Dementia Strategy emphasizes the importance of this telephone advice service [19].

So far, however, no research has been carried out into whether the Alzheimer’s telephone meets the expectations and needs of those seeking advice. In this paper, we report on the evaluation of the ATS. The evaluation was based on an online survey of 201 users after the telephone consultation. Suggestions for improvement are to be derived from the results, which can also be useful for similar consulting services.

## 2 Methods

For the survey of global user ratings, the patient satisfaction questionnaire (ZUF-8) [20] was adapted to the context of telephone consultations. The sum of the 8 four-point scale items ranges from 8 (lowest satisfaction) to 32 (highest satisfaction). In the absence of a suitable German-language instrument, we developed our own questionnaire to assess individual aspects of counseling (see Attachment 1) [21]. On the basis of the quality model in the health care sector [22], the counseling service is evaluated with a total of 13 5-level scaled items regarding structural conditions (accessibility of the Alzheimer’s telephone), the counseling process (counselor behavior, appreciation, time flexibility, empathy, precise recording of the concern, dealing with the limits of one’s own knowledge, solution-oriented conversation, professional competence) and the outcome of the conversation (quality of the information received, indication of the next steps for action, practical feasibility of the proposed solutions, contribution to overcoming the current problem). In a free text field, the study participants were also able to make suggestions and proposals for improvements to the ATS. The data were collected online between February 1^st^, 2022, and April 28^th^, 2022, using the Limesurvey application. Immediately after the telephone consultation, callers who had given their consent were given access to the questionnaire by email. The study included relatives or unrelated caregivers of a person with dementia as the main target group of the ATS. Persons with dementia and professionals in the field of dementia care were not included in the study. The study has been approved by the ethics committee of the Ludwig-Maximilians-Universität (project number 23-0316 KB). Group differences in metric variables were calculated using a simple analysis of variance. Differences in the distribution of ordinally scaled variables were calculated using the Kruskal-Wallis test. A Bonferroni correction for multiple testing was applied. Effect sizes between nominally scaled and metric variables were calculated using the eta coefficient, and effect sizes between nominally and ordinally scaled variables were calculated using the phi coefficient. Effect sizes were classified as small (0.1), medium (0.2), and large (0.3) [23]. Data were analyzed using the Statistical Package for the Social Sciences (SPSS) version 27. 

## 3 Results

### Description of the users

**Figure 1:**
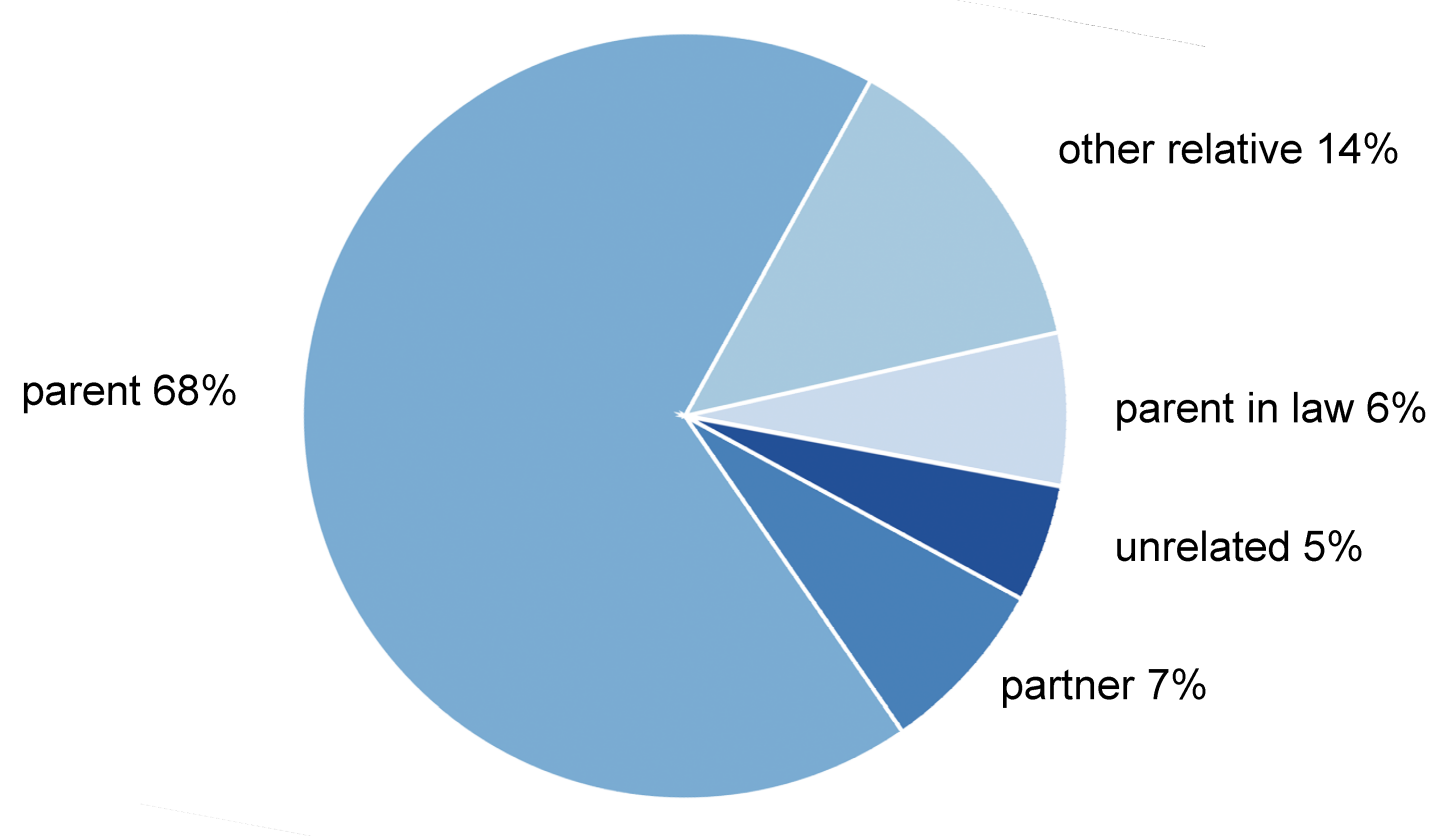
Relationship to person with dementia

**Figure 2:**
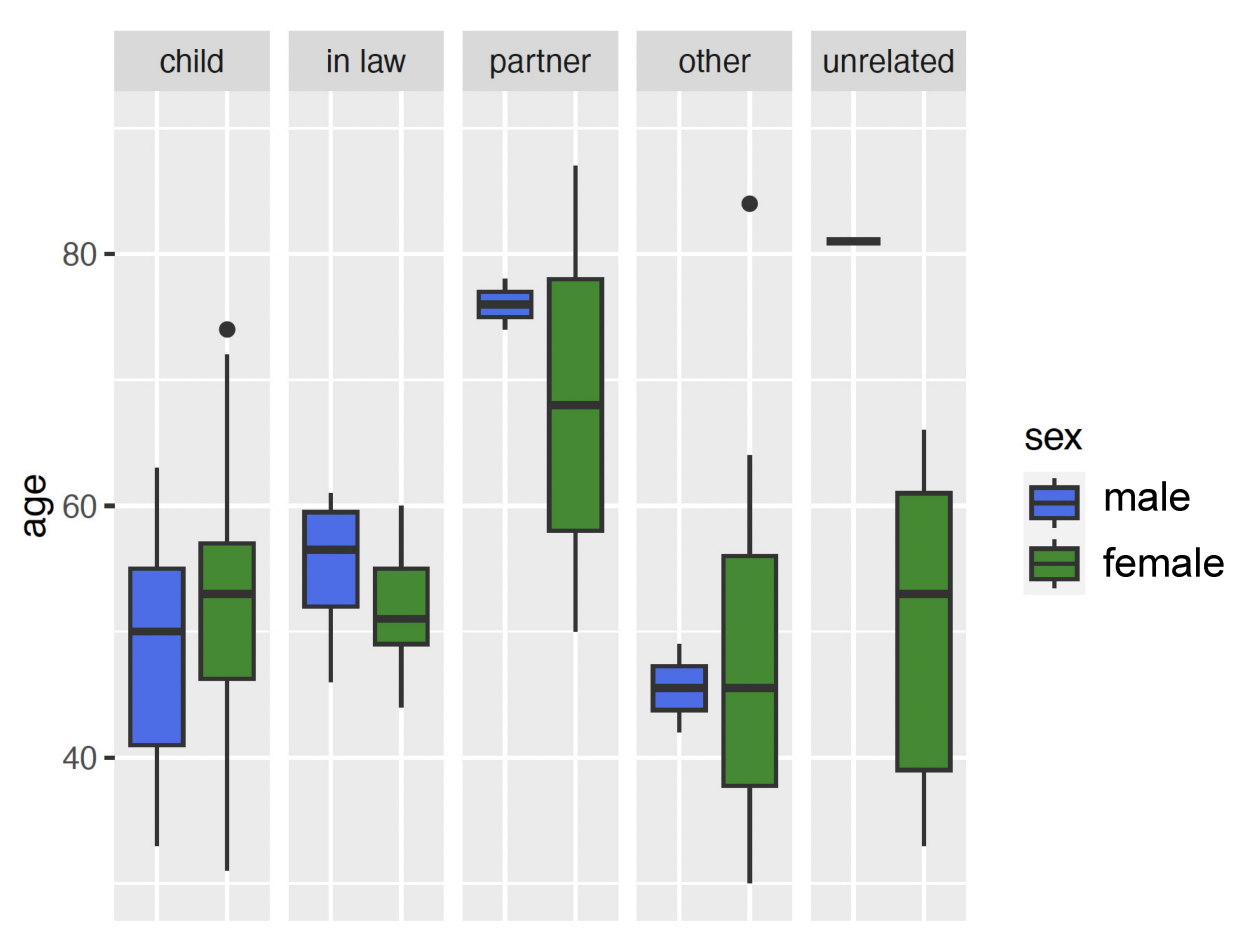
Age and gender distribution of callers

A total of 205 callers participated in the online survey. Four of them were excluded because they stated that they had contacted the Alzheimer’s helpline for educational purposes. There were five groups of callers: children, children-in-law, spouses and partners, other relatives and unrelated persons. Children or children-in-law were the largest group of relatives, accounting for about three-quarters of callers (74%), followed by other relatives (13%), spouses or partners (8%), and unrelated persons (5%) (Figure 1 (Fig. 1)). Of the remaining 201 participants, 80% were female and the mean age was 51 years (Table 1 (Tab. 1), Figure 2 (Fig. 2)).

The partners of the person with dementia were significantly (p<0.001) older (mean age 69 years) than the children(-in-law) (mean age 51 or 52 years) or other relatives (mean age 42 years) or unrelated persons (mean age 54 years). There were no significant differences in the gender distribution between the individual caller groups (Figure 2 (Fig. 2)). Approximately half (47%) of the participants were the primary caregiver of the person with dementia, with spouses and partners being significantly more likely (p<0.001) to be the primary caregiver of the person with dementia. The living situation of the callers varied: 91% did not live in the same household as the person with dementia; spouses were significantly (p<0.001) more likely to live with the person with dementia. The frequency of personal contact with the person with dementia was reported as daily by 22% of callers, once or several times a week by 36%, and once a month or less by 31%. (Marital) partners had significantly (p<0.001) more frequent contact with the person with dementia than other relatives and non-relatives. Academics (49%) made up nearly half of the callers across all groups.

### Reasons for reaching out

**Figure 3:**
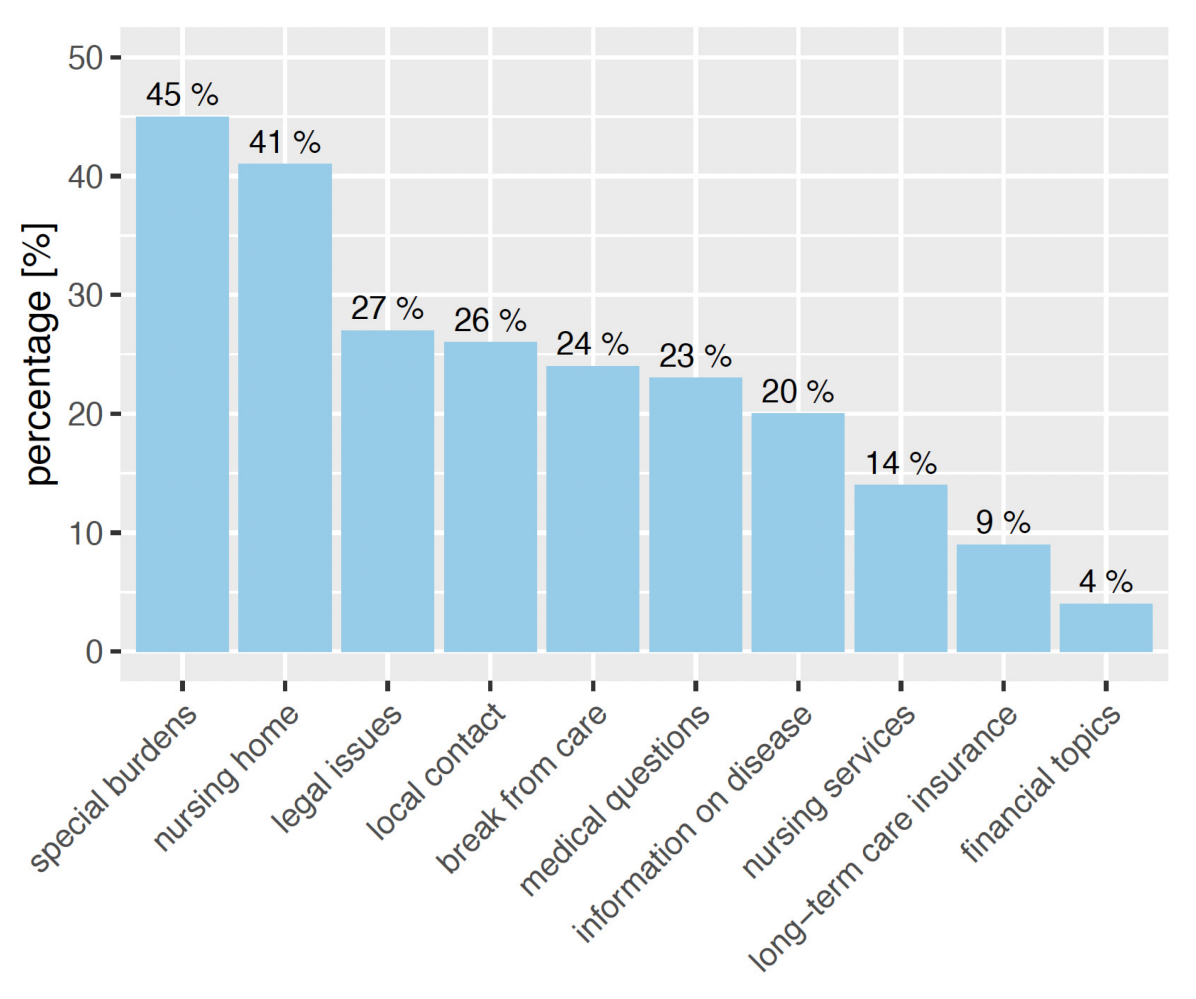
Reasons for the call (multiple answers; N=201; figures in [%])

The most common reasons for calling the Alzheimer’s disease hotline were related to the particular burden on family members. These included pronounced behavioral changes (45%) and finding a nursing home (41%). Legal issues (27%), finding local contacts (26%), and medical issues (26%) were less frequently mentioned (Table 2 (Tab. 2), Figure 3 (Fig. 3)). Financial issues (4%), questions about long-term care insurance (9%), and outpatient care (14%) were less common reasons for contact. Other family members were significantly (p=0.036) more likely to seek local respite options. 

### Structural conditions

Most callers (74%) had one conversation, while partners were significantly (p=0.042) more likely to have two conversations. The duration of the consultation was 17 to 40 (25^th^ and 75^th^ percentile) minutes with an average of 29 minutes. Regarding the duration of the counseling session, there were no differences between the groups of callers (Table 3 (Tab. 3)). More than two thirds (69%) of the callers needed only one call attempt to reach a counselor. 14% needed two calls and 17% needed more calls to reach a counselor within a reasonable waiting time. Overall, 93% were satisfied or very satisfied with the availability of the ATS and the majority (93%) of callers would contact the ATS again (Table 4 (Tab. 4)).

### Overall advice

The ZUF-8 showed an overall high level of satisfaction, with an average overall score of 30 out of a maximum of 32 (Table 4 (Tab. 4)). In each case, 99% of respondents said they would contact the ATS again if they had a problem and would recommend it to a friend in a similar situation. Only 0.5–1% would not do so. 2% of callers indicated that the advice they received did not meet their needs and that they were not satisfied with the level of assistance they received. More than 98% of the study participants were fairly satisfied and very satisfied (ZUF-8>24). The callers who were not fully satisfied (n=9) tended to be younger, with a mean age of 45 years (p=0.05), and had contacted the ATS more often for a problem other than the main questions listed above (Table 5 (Tab. 5)).

### Assessment of the process quality

Participants’ ratings of the process and atmosphere were overwhelmingly positive. Overall, most callers in all caller groups were satisfied with the quality of the counseling session, the help offered, and the type of counseling (Table 6 (Tab. 6)). More than 90% of callers gave the highest marks to counselor appreciation, time spent, empathy, and accuracy of problem assessment. However, some of the participants were not quite as convinced about the focus of the conversation and the transparent presentation of the possibilities and limitations of telephone counseling. 20% of callers felt that the counselor did not fully answer their questions. There were no differences between the caller groups in terms of satisfaction with the counseling process.

### Assessment of the quality of results at the factual level

Most callers reported that the counselors were empathetic and appreciative. 86% of callers strongly agreed that the information they received was helpful (Table 7 (Tab. 7)). For 75% of callers, the helpline indicated the next steps for them, with only 5% undecided or doubtful that the helpline had given them more competence to act. 

### Assessment of the quality of results on an emotional level

About half of the callers (48%) expressed full confidence that the proposed solutions were feasible for them and would help solve the problem at hand. Most callers also reported feeling relieved after the consultation (Table 7 (Tab. 7)). 30% said they felt more confident in dealing with the person with dementia after the consultation. Negative feelings such as uncertainty, hopelessness or stress were overwhelmingly denied in all groups of callers. Callers felt that the counseling was appropriate and appreciated the counsellor’s balancing act between too personal and too impersonal consultation (Table 8 (Tab. 8)).

## 4 Discussion

Family caregiver telephone counseling is an easily accessible and free opportunity to support family caregivers of a person with dementia. Counseling family members of a person with dementia is extremely complex given the many areas of life that can change as dementia progresses. The research project aimed to find out whether the ATS meets caregivers’ needs, what constitutes good telephone counseling, and when counseling can be considered successful.

From the users’ perspective, the results of the study paint a very positive picture of the counseling provided by the ATS. Most respondents said that the quality of the counseling was excellent, that the information they received was helpful, and that they would recommend the telephone counseling service to others and use it again if necessary. Participants also highly valued the structural features of the helpline, such as its accessibility, as well as most aspects of the way the counselors conducted the conversation.

The study also highlights the challenges of a dementia helpline. To be able to deal with the wide range of questions and problems that the counselors are asked to deal with, a comprehensive medical, psychological, socio-pedagogical and legal qualification as well as economic background knowledge is required, which can hardly be provided by a single staff member. It is therefore surprising that the counselors were generally able to meet these high standards. Only in a few cases were they unable to fully meet the needs of the callers. 

Ongoing supervision, case discussions, and regular meetings within the counseling team appear to be important in maintaining such a high level of counseling. The ATS team has also worked continuously on a digital database since its inception. This is still being updated with new information, especially on rare and challenging consultation requests.

Good counseling is characterized by the right balance between too much and too little information, too distant or too personal communication, overstepping boundaries of competence, and hasty referrals. Counselors on the ATS were generally successful in maintaining this balance. The aim of the advice given on the Alzheimer’s helpline was often to show the caller a way of dealing with a current, often complex problem and to point out the next step to be taken. In this way, the main goal is to strengthen the self-help skills of the callers and to increase their self-efficacy.

When assessing the quality of the results on an emotional level, doubts were expressed about the extent to which the suggestions could be put into practice and contribute to solving the problem. And yet, it is precisely this uncertainty that can provide another clue as to how the counseling service can be improved: how can the counseling of clients be more effectively supported in the implementation of the proposed solutions, in the mobilization of support resources at home, in the involvement of the necessary cooperation partners, or in switching to an alternative strategy if the path taken so far proves to be unfeasible? On the one hand, it is useful to suggest a follow-up meeting and, on the other hand, to indicate the possibility of a personal consultation at the place of residence. In addition, the long and growing experience of the Alzheimer’s helpline staff could be passed on to the regional counselors in training sessions. In the long run, this could lead to a nationwide counseling network with common strategies and goals. However, the burden of implementing the advice given lies with the caller. In addition, the barriers to the implementation of the counseling for the family caregivers should be investigated.

Some limitations of the present study should not go unmentioned:

The proportion of women in our sample (80%) was slightly higher than in a recent national survey of family caregivers (72%), meaning that the concerns of male family caregivers of people with dementia are underrepresented in this analysis [24]. User data from the Alzheimer’s telephone confirms that the majority of informal care for people with dementia is provided by women [25].

Although the online survey format made it possible to reach many callers with little effort, this method excluded caregivers who did not have an email address from participating. This is a possible explanation for the facts that the study participants were relatively young, with an average age of 51, and that the vast majority did not live in the same household as the person with dementia. It may be that the advice provided by the Alzheimer’s helpline is valued differently by older relatives and (marital) partners than by relatives providing care from a distance. The digital survey application, which older participants may have reservations about, may have led to a bias effect. The present evaluation showed that the less satisfied callers tended to be younger people who might be looking for a different kind of advice. However, important influencing variables such as the type of dementia or further information on the clinical picture, a migration background, the duration of care or the professional activity of the relatives were not collected [4], [5], [26]. This information should be obtained as part of a further research project in order to adjust the advice more specifically. The technical medium of communication, i.e. the telephone, was also not addressed. The need for additional services such as online video conferencing or tutorials was not asked about.

In summary, users gave the most widespread telephone helpline for family members of people with dementia in Germany a very good rating. They were highly satisfied with the organizational aspects of the Alzheimer’s helpline, the communication skills of the counselors, their professional competence, the quality of the information received, and the usefulness of the counseling session. The positive evaluation shows that the advice provided by the Alzheimer’s helpline is a useful component of dementia care in Germany that can be implemented in everyday life and is an important contribution to the National Dementia Strategy. The results of this study can help to maintain and improve this important service.

## Key messages

- The National Dementia Strategy emphasizes personal advice over the phone as an important source of help for family caregivers of people with dementia.
- One such service is the Alzheimer’s Telephone Service of the German Alzheimer’s Society, which has been in existence since 2002 and offers free advice on all aspects of dementia throughout Germany.
- In an online survey of 201 users, the extent to which this service meets the quality criteria of telephone help services and the needs of callers was examined.
- Most participants were very satisfied with the accessibility and quality of the advice, the communication style of the advisors and the help they received.
- Telephone counseling is a useful part of dementia care and can be recommended to relatives of people with dementia seeking advice.
- The nature of the evaluation using a digital questionnaire should be reconsidered as it may have had a bias effect on the participants.
- The aim of the call as well as the possibilities and limitations of telephone counseling should be communicated transparently.
- General practitioners should be informed about the counseling service.
- Services for special groups of caregiving relatives (younger relatives) should be expanded.
- Information about people with dementia should be specifically collected as part of a further research project to better target needs.
- A national counseling network for carers should be established with common strategies and goals.

## Abbreviations

- ATS: Alzheimer’s telephone service
- CI: confidence interval
- n.a.: not applicable
- n: number
- r: effect size
- SD: standard deviation

## Notes

### Acknowledgements and funding

This work is dedicated to Professor Dr. med. Alexander Kurz (1950–2023), who unfortunately did not live to see the completion of this scientific work.

We would like to thank Helga Schneider-Schelte for her support in creating the questionnaire and Lea Pfäffel for proofreading the manuscript. The evaluation of the Alzheimer’s telephone was funded by the Porticus Foundation and was carried out by a multiprofessional team at the Department of Psychiatry and Psychotherapy, Technical University of Munich.

### Ethics statement

The study was approved by the ethics committee of the Ludwig-Maximilians-Universität, Munich, Germany (project number 23-0316 KB).

### Competing interests

AK was a member of the board of the German Alzheimer’s Society until his death. IMF, VD and CK declare that they have no competing interests. 

## Supplementary Material

Alzheimer’s telephone user survey

## Figures and Tables

**Table 1:**
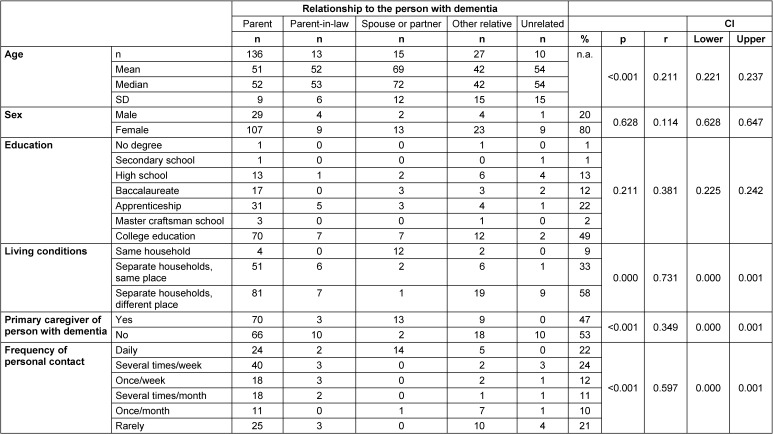
Description of the callers

**Table 2:**
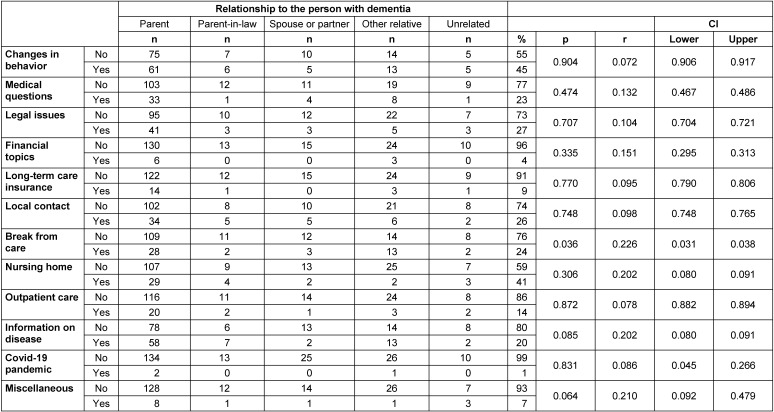
Reasons for calling (multiple answers possible, n=201)

**Table 3:**
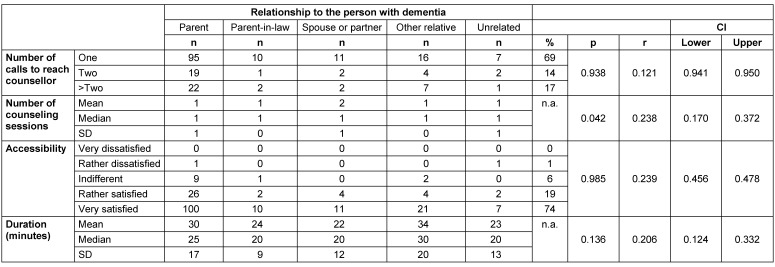
Structural conditions

**Table 4:**
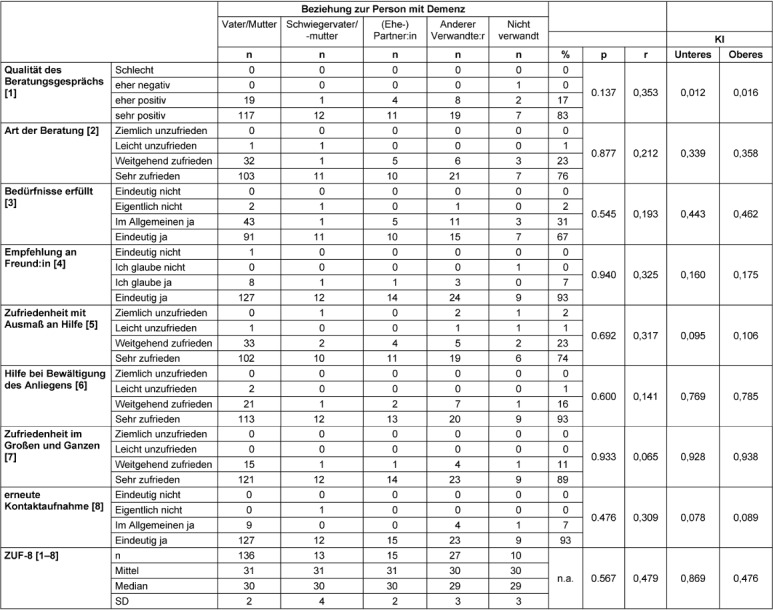
Satisfaction with counseling session

**Table 5:**
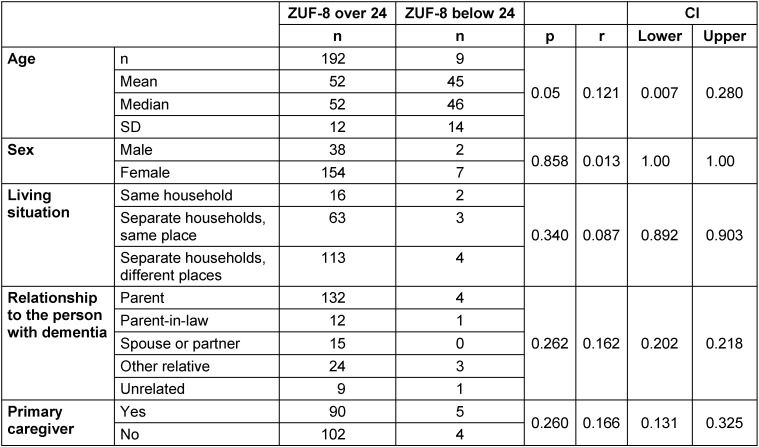
Characterization of not fully satisfied callers

**Table 6:**
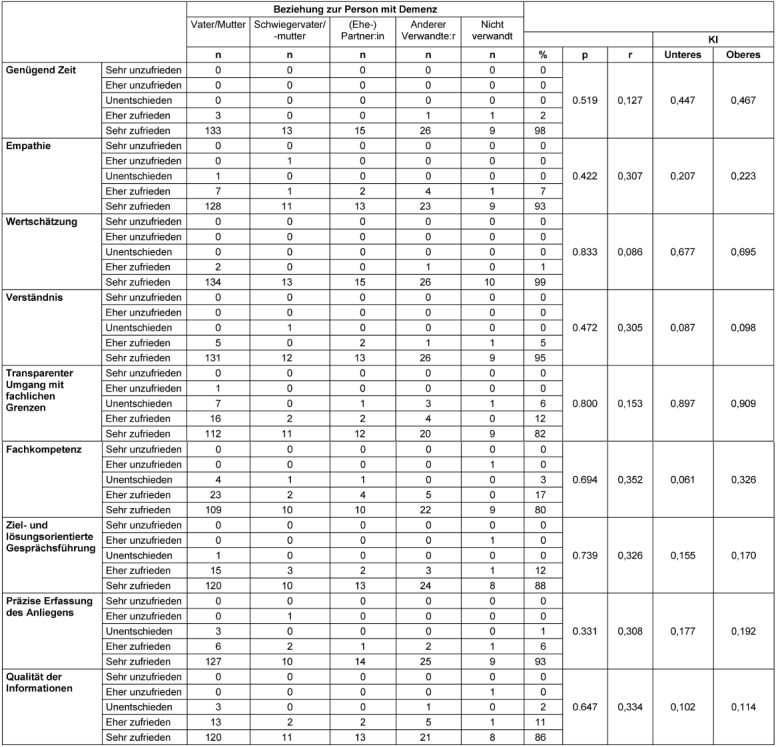
Assessment of process quality (n=201)

**Table 7:**
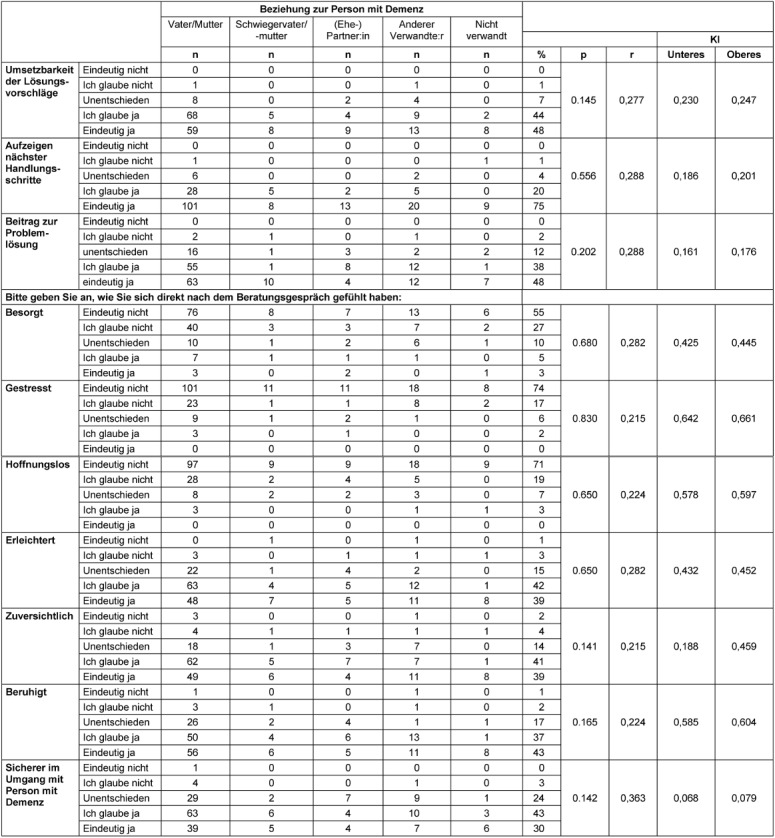
Assessment of the quality of results (n=201)

**Table 8:**
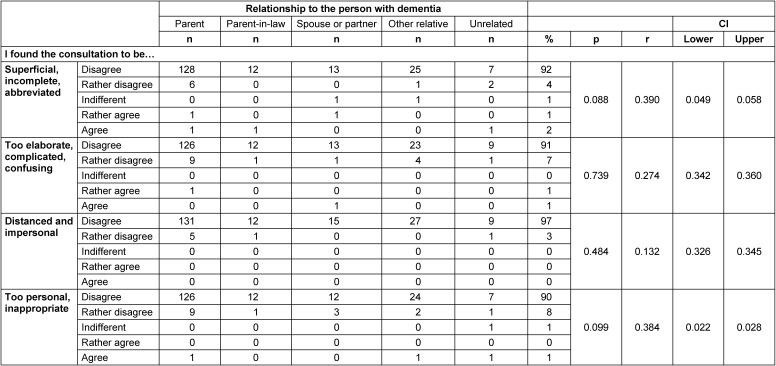
Reasons for dissatisfaction [%]
