# Supplementary material for: Self-help support: The Alzheimer’s telephone from the user’s perspective
Source: Ger Med Sci. 2024 Apr 24;22:Doc05. doi: 10.3205/000331 (PMC11176817; doi:10.3205/000331)
Supplement: Alzheimer’s telephone user survey [file GMS-22-05-s-001.pdf]

Alzheimer’s telephone user survey

Dear participant,

With your consent, the **German Alzheimer’s Society** has informed us that you have recently taken advantage of a consultation via the “Alzheimer’s telephone”. With your help we – a team of researchers at the Klinikum rechts der Isar of the Technical University of Munich – would like to gain insights into the quality of this advisory service from your perspective.

Please read the instructions for the questionnaire carefully and try to answer all questions honestly and spontaneously. What matters to us is your personal assessment. There are no right or wrong answers.

Participation in our study is of course voluntary. You can end your participation at any time without giving reasons or refuse to participate altogether. All information is collected and processed **anonymously.** It is not possible to identify you personally.

Please take about 10 minutes to complete the questionnaire alone in a quiet environment.

If you have any questions or are interested in the results of the survey, please do not hesitate to contact us.

By clicking on “Continue”, you give your consent to participate in this survey and to the anonymized processing of your data.

Thank you for your time and support!

This survey contains 38 questions.

Please provide some information about yourself:

How old are you? *

Please enter your answer here:

Your gender: *

Please select only one of the following answers:

- ◯ Male
- ◯ Female
- ◯ Diverse

What is your highest educational qualification? *

Please select only one of the following answers:

- ◯ No degree
- ◯ Secondary school
- ◯ High school
- ◯ Baccalaureate
- ◯ Apprenticeship
- ◯ Craftsman school
- ◯ College education

Please provide some information about the person with dementia who was the subject of your last consultation with the Alzheimer’s helpline

The person with dementia who was the subject of the consultation with the Alzheimer's hotline is my *.

Please select only one of the following answers:

- ◯ Father/mother
- ◯ Parent-in-law
- ◯ (Marital) partner/partner
- ◯ Son/daughter
- ◯ Other relative
- ◯ Not related to me (friend, acquaintance, neighbor, etc.)
- ◯ I am person with dementia

I see the person with dementia... *

Please select only one of the following answers:

- ◯ Daily
- ◯ Several times a week
- ◯ Once a week
- ◯ Several times a month
- ◯ Once a month
- ◯ Less often

I am the primary caregiver of the person with dementia *

Please select only one of the following answers:

- ◯ Yes
- ◯ No

I live with the person with dementia... *

Please select only one of the following answers:

- ◯ In the same household
- ◯ In separate households but in the same place
- ◯ In separate households and in different locations

I contacted the Alzheimer’s hotline during my last call for the following reason(s): (multiple answers possible) *

Please select all applicable answers:

- ☐ Questions regarding how to deal with the person with dementia
- ☐ Changes in behavior
- ☐ Medical question (incl. doctor search)
- ☐ Legal question
- ☐ Financial issues
- ☐ Long-term care insurance
- ☐ Search for a local contact person
- ☐ Search for relief options
- ☐ Information on the clinical picture
- ☐ Outpatient (care) services
- ☐ Covid-19
- ☐ Other
- ☐ Nursing home

Please use a keyword to indicate the subject of your call:

Please enter your answer here:

How many consultations with the Alzheimer’s telephone, including the last consultation, have you already had? *

Please select only one of the following answers:

- One
- two
- More than two

How many times did you have to call before you reached an advisor? *

Please select only one of the following answers:

- Once
- Twice
- More than twice

I am not happy with the accessibility of the Alzheimer’s telephone... *

Please select only one of the following answers:

- ◯ Very satisfied
- ◯ Satisfied
- ◯ Undifferent
- ◯ Dissatisfied
- ◯ Very dissatisfied

Why are you undecided or dissatisfied about the availability of the Alzheimer’s telephone?

*

Please enter your answer here:

Please estimate the duration of the last consultation with the Alzheimer’s telephone in minutes

*

Please enter your answer here:

The consultant had enough time for me *

Please select only one of the following answers:

- ◯ Fully agree
- ◯ Rather agree
- ◯ Indifferent
- ◯ Rather disagree
- ◯ Strongly disagree

I had the feeling that the counselor knew how I felt *

Please select only one of the following answers:

- ◯ Fully agree
- ◯ Rather agree
- ◯ Indifferent
- ◯ Rather disagree
- ◯ Strongly disagree

The counselor treated me with respect *

Please select only one of the following answers:

- ◯ Fully agree
- ◯ Rather agree
- ◯ Indifferent
- ◯ Rather disagree
- ◯ Strongly disagree

I felt understood by the counselor *

Please select only one of the following answers:

- ◯ Fully agree
- ◯ Rather agree
- ◯ Indifferent
- ◯ Rather disagree
- ◯ Strongly disagree

The advisor communicated the limits of the advice transparently *

Please select only one of the following answers:

- ◯ Fully agree
- ◯ Rather agree
- ◯ Indifferent
- ◯ Rather disagree
- ◯ Strongly disagree

The consultant was able to answer my questions *

Please select only one of the following answers:

- ◯ Fully agree
- ◯ Rather agree
- ◯ Indifferent
- ◯ Rather disagree
- ◯ Strongly disagree

The counselor proceeded in a goal- and solution-oriented manner *

Please select only one of the following answers:

- ◯ Fully agree
- ◯ Rather agree
- ◯ Indifferent
- ◯ Rather disagree
- ◯ Strongly disagree

The consultant understood my concerns precisely *

Please select only one of the following answers:

- ◯ Fully agree
- ◯ Rather agree
- ◯ Indifferent
- ◯ Rather disagree
- ◯ Strongly disagree

The information I received was helpful *

Please select only one of the following answers:

- ◯ Fully agree
- ◯ Rather agree
- ◯ Indifferent
- ◯ Rather disagree
- ◯ Strongly disagree

I am confident that I will be able to implement the solutions I have received *

Please select only one of the following answers:

- ◯ Fully agree
- ◯ Rather agree
- ◯ Indifferent
- ◯ Rather disagree
- ◯ Strongly disagree

The consultation showed me the next step *

Please select only one of the following answers:

- ◯ Fully agree
- ◯ Rather agree
- ◯ Indifferent
- ◯ Rather disagree
- ◯ Strongly disagree

The consultation helped to resolve my concerns *

Please select only one of the following answers:

- ◯ Fully agree
- ◯ Rather agree
- ◯ Indifferent
- ◯ Rather disagree
- ◯ Strongly disagree

I found the consultation to be...

*

Please select the appropriate answer for each item:

	Fully agree	Rather agree	Indifferent	Rather disagree	Fully disagree
**Too superficial, incomplete, abbreviated**	○	○	○	○	○
**Too detailed, complicated, confusing**	○	○	○	○	○

I found the consultant to be...

*

Please select the appropriate answer for each item:

	Fully agree	Rather agree	Indifferent	Rather disagree	Fully disagree
**Too distant and cool**	○	○	○	○	○
**Too personal, friendly, inappropriate**	○	○	○	○	○

Please indicate how you generally perceived the consultation:

How would you rate the quality of the consultation with the Alzheimer’s hotline in general? *

Please select only one of the following answers:

- ◯ Excellent
- ◯ Good
- ◯ Less good
- ◯ Bad

Did you get the kind of advice you wanted? *

Please select only one of the following answers:

- ◯ Definitely not
- ◯ Actually not
- ◯ Generally yes
- ◯ Definitely yes

To what extent did the consultation meet your needs? *

Please select only one of the following answers:

- ◯ It has met almost all of my needs.
- ◯ It has met most of my needs.
- ◯ It has met only a few of my needs.
- ◯ It has not met my needs.

Would you recommend the Alzheimer’s telephone to a friend if he/she needed similar help? *

Please select only one of the following answers:

- ◯ Definitely not
- ◯ I don’t think so
- ◯ I think so
- ◯ Definitely yes

How satisfied are you with the amount of help you have received? *

Please select only one of the following answers:

- ◯ Fairly dissatisfied
- ◯ Slightly dissatisfied
- ◯ Largely satisfied
- ◯ Very satisfied

Did the consultation help you to deal with your concerns? *

Please select only one of the following answers:

- ◯ Yes, it helped a lot.
- ◯ Yes, it helped a little.
- ◯ No, it didn’t actually help.
- ◯ No, it made things harder for me.

Overall, how satisfied are you with the advice you received? *

Please select only one of the following answers:

- ◯ Very satisfied
- ◯ Largely satisfied
- ◯ Slightly dissatisfied
- ◯ Fairly dissatisfied

Would you contact the Alzheimer’s hotline again if you needed help? *

Please select only one of the following answers:

- ◯ Definitely not
- ◯ I don’t think so
- ◯ I think so
- ◯ Definitely yes

Please indicate how you felt immediately after your consultation with the Alzheimer’s helpline: *

Please select the appropriate answer for each item:

	Fully agree	Rather agree	Indifferent	Rather disagree	Fully disagree
**Worried**	○	○	○	○	○
**Stressed**	○	○	○	○	○
**Hopeless**	○	○	○	○	○
**Facilitates**	○	○	○	○	○
**Confident**	○	○	○	○	○
**calms**	○	○	○	○	○
**More confident in dealing with people with dementia**	○	○	○	○	○

Do you have any suggestions, requests or other feedback for us regarding the Alzheimer’s telephone?

Please enter your answer here:

Thank you very much for your participation!

They have helped to record satisfaction with the “Alzheimer’s telephone”.
